# Genetic engineering: an efficient approach to mitigating biotic and abiotic stresses in sugarcane cultivation

**DOI:** 10.1080/15592324.2022.2108253

**Published:** 2022-08-12

**Authors:** Krishan K. Verma, Xiu-Peng Song, Florencia Budeguer, Amin Nikpay, Ramon Enrique, Munna Singh, Bao-Qing Zhang, Jian-Ming Wu, Yang-Rui Li

**Affiliations:** a Sugarcane Research Institute, Guangxi Academy of Agricultural Sciences/ Sugarcane Research Center, Chinese Academy of Agricultural Sciences/ Key Laboratory of Sugarcane Biotechnology and Genetic Improvement (Guangxi), Ministry of Agriculture and Rural Affairs/ Guangxi Key Laboratory of Sugarcane Genetic Improvement, Nanning, China; b Instituto de Tecnología Agroindustrial del Noroeste Argentino (ITANOA), Estacion Experimental Agroindustrial Obispo Colombres (EEAOC) – Consejo Nacional de Investigaciones Científicas y Técnicas (CONICET), Las Talitas, Argentina; c Department of Plant Protection, Sugarcane and By-Products Development Company, Salman Farsi Agroindustry, Ahwaz Iran; d Department of Botany, University of Lucknow, Lucknow – India

**Keywords:** Biotic and abiotic stress resistance, genetic engineering, stress reliever, transformation approaches, sugarcane (*saccharum* spp.)

## Abstract

Biotic and abiotic stresses are the foremost limiting factors for crop productivity. Crop plants need to cope with adverse external pressure caused by various environmental conditions with their intrinsic biological mechanisms to keep their growth, development, and productivity. Climate-resilient, pest resistance and high-yielding crops need to be developed to maintain sustainable food supply. Over the last decade, understanding of the genetic complexity of agronomic traits in sugarcane has prompted the integrated application of genetic engineering to address specific biological questions. Genes for adaptation to environmental stress, resistance to pest and yield enhancement traits are being determined and introgressed to develop elite sugarcane cultivars with improved characteristics through genetic engineering approaches. Here, we discuss the advancement to provide a reference for future sugarcane (*Saccharum* spp.) genetic engineering.

## Introduction

Sugarcane (*Saccharum* spp.) is one of the major industrial crops in the world due to sugar being regarded as a strategic food commodity because of its importance in meeting the calorie needs of people and for the commercial food and beverage industries.^1^ New tasks include successfully utilizing sugarcane as a renewable bioenergy crop. The approaches of genetic and biotechnological advancement have been applied in the selection of superior and all-purpose cultivars for agro-industries. Sugarcane production is negatively affected by various biotic stresses (diseases, pests and weeds) and abiotic stresses (low or excessive water, high salinity, high or low temperatures, heavy metals, ultraviolet radiation, wind, and low soil fertility), reducing the productivity of the crops.^2,3^ For this reason, conventional breeding supported by genetic engineering constantly seeks to develop new genotypes with high sugar and biomass and strong stress tolerance. However, advancing natural genetic resources to generate varieties with resistance is a big challenge. In this sense, elite varieties are the starter materials for crop improvement through genetic engineering to add traits that confer adaptative advantages to overcome the stresses mentioned.

Genetic transformation (GT) and genome editing (GE) are the most valuable biotechnological tools. The first technology makes it possible to incorporate genes from unrelated plants or different organisms. Meanwhile, GE can edit, insert, or replace specific sequences within the genome.^4^ Several genes for resistance to biotic and abiotic stresses have been introduced into sugarcane to improve the crop.^5,6^ The success of producing transgenic sugarcane plants depends on the method used for transformation, the use of strong promoters in the transformation vectors, the target tissue/explants, the type of selection, and the tissue culture regeneration system (Table 1). For that reason, serious attention to enhancing sugarcane productivity through genetic engineering has been carried out during the last decades in various sugarcane-growing countries around the globe.

**Table 1.** Genetic engineering of sugarcane for different traits.

Type of stress	Trait	Gene	Gene function	Transformation method	Sources
Biotic stress	Herbicide resistance	*bar*	Confers resistance to bialaphos	Biolistic	^[7–10]^
		*EPSPS*	Confers resistance to glyphosate	Biolistic	^[11–13]^
		*als*	Confers resistance to ALS inhibitors	Biolistic	^[14]^
		*bar*	Confers resistance to phosphinothricine	*Agrobacterium/ Microprojectile*	^[15]^
		*pat*	Confers resistance to glufosinate ammonium	*Microprojectile*	^[16]^
	Disease resistance	*CP*	Prevents various stages of the viral life cycle	Biolistic/*Agrobacterium*	^[17–20]^
		*CP*	Prevents various stages of the viral life cycle	Biolistic/*Agrobacterium*	^[21,22]^
		*CP*	Prevents various stages of the viral life cycle	Biolistic	^[23,24]^
		*Alb D*	Albicidin detoxification	Biolistic	^[25]^
		*β-1,3 glucanase*	Degradation of the fungal cell wall	*Agrobacterium*	^[26]^
		*Chitinase Class II*	Degradation of the fungal cell wall	Biolistic	^[27]^
	Pest resistance	*cry1Ab*	Confers resistance to lepidopteran by selectively damaging their midgut lining	Electroparation/Biolistic *Agrobacterium*	^[11,28–33]^
		*cry1Ac*	Confers resistance to lepidopteran by selectively damaging their midgut lining	Biolistic/*Agrobacterium*	^[34–39]^
		*cry1Aa3*	Confers resistance to lepidopteran by selectively damaging their midgut lining	*Agrobacterium*	^[40]^
		*cry2A*	Confers resistance to lepidopteran by selectively damaging their midgut lining	Biolistic	^[41]^
		*Vip3A*	Confers resistance to lepidopteran by selectively damaging their midgut lining	*Agrobacterium*	^[42]^
		*cry1Ab and cry2Ab*	Confers resistance to lepidopteran by selectively damaging their midgut lining	*Agrobacterium*	^[43]^
		*CryIAb-CryIAc*	Confers resistance to lepidopteran by selectively damaging their midgut lining	*Agrobacterium*	^[44]^
		*SKTI & SBBI*	Inactivate digestive proteases and decrease growth in Lepidoptera	Biolistic	^[45]^
		*AVAc-SKTI*	Inactivate digestive proteases and decrease growth in Lepidoptera	*Agrobacterium*	^[46]^
		*HIS Cane CPI-1*	Inactivate digestive proteases and decrease growth in Lepidoptera	Biolistic	^[47,48]^
		*aprotinin*	Inactivate digestive proteases and decrease growth in pest	Biolistic	^[49]^
Abiotic stress	Drought	*Tsase*	Biomolecules stabilization	*Agrobacterium*	^[50]^
		*AVP1*	Osmotic regulation	*Agrobacterium*/Biolistic	^[51,52]^
		*DREB2A CA*	Gene regulation	Biolistic	^[53]^
		*BI-1*	Program cell death regulation	Biolistic	^[54]^
		*SoP5CS*	Proline synthesis	*Agrobacterium*	^[55]^
		*AtBBX29*	Gene regulation	Biolistic	^[56]^
		*TERF1*	Gene regulation	*Agrobacterium*	^[57]^
	Salinity	*P5CS*	Proline synthesis	Biolistic	^[58]^
		*HSP70*	Celular components; stabilization	*Agrobacterium*	^[59]^
		*EaGly III*	Reduce oxidative damage	Biolistic	^[60]^
	Cold	*ipt*	Cytoquinin synthesis	Biolistic	^[61]^
		*SoTUA*	α-tubulin synthesis	*Agrobacterium*	^[62]^

## Biotic stress – disease and pest resistance

Sugarcane cultivation is significantly influenced by various biotic stressors, and is the main reason for the fluctuation of sugar production. In sugarcane growth and development, diseases, pests, nematodes, and weeds are major biotic stressors affecting overall the plant performance.

Disease control requires an integrated approach involving the use of disease-resistant cultivars and disease-free materials, and establishing strict quarantine measures.^2^ More than 100 pathogens that cause diseases in sugarcane are known, including bacteria, fungi, viruses, phytoplasmas and nematodes.^63,64^ It is challenging in breeding programs to introduce the germplasm with high productivity, high sucrose content, strong resistance to biotic and abiotic stresses, and enhanced rationing ability through conventional breeding since wide varieties are susceptible to more than one pathogen.^3^ Therefore, researchers focus on studying new biotechnological breeding strategies for commercial clones with high agronomic performance.^65^

Mosaic and yellow leaf syndrome are the two most important viral diseases in sugarcane globally. Mosaic disease is caused by sugarcane mosaic virus (SCMV) and sorghum mosaic virus (SrMV)^66,67^ and yellow leaf syndrome, is caused by sugarcane yellow leaf virus (SCYLV). Worldwide economic losses were reported due to the both diseases, so genetic transformation strategies have been implemented to obtain plants resistant to these diseases.^67^ The first work about obtaining virus-resistant transgenic plants used the virus capsid protein (CP). These plants expressed a homolog of a viral protein, bypassing various stages of the virus life cycle and attenuating the disease symptom.^21^ RNA interference (RNAi)-mediated defense against viral infection was found to be a major innate immune response. As a counter attack strategy in response to the host defense, viruses produce suppressors of host RNAi pathway. Identification of miRNAs encoded by sugarcane streak mosaic virus and understanding their host target genes might be used as a new strategy to study viral pathogenesis and controlling mosaic disease in sugarcane.^68^

The first development of a virus-resistant transgenic sugarcane plant was described by Joyce et al.^17^ (1998). They transformed sugarcane explants with the *CP* gene by microprojectile bombardment and obtained ten transgenic lines resistant to sugarcane mosaic potyvirus (SCMV). Then, Ingelbrecht et al.^21^ (1999) developed transgenic sugarcane plants derived from an untranslatable form of the *CP* gene from a SrMV strain. The transgenic plants showed a variable response when challenged with the virus, ranging from fully susceptible to fully resistant in phenotype. On the other hand, Apristi et al.^18^ (2018) compared the resistance to SCMV virus in the transformed plants with the complete sequence of the *CP* SCMV gene (927 base pairs, bp) and those with the truncated sequences (702 bp), and found more excellent protection against the virus in the plants transformed with the complete gene sequence. Regarding the studies that used RNA interference technology to obtain virus-resistant plants, it was observed that the plants expressed short hairpin RNAs (shRNA) showed immunity to SCMV infection.^19,22^

Phenotypic changes in the population of 100 transgenic plants were extremely high, emphasizing the need for thorough field evaluation of transgenic sugarcane. However, the large variability in the transgenic materials allowed for the identification of several transgenic accessions with improved growth and yield characteristics, and disease resistance compared with the commercial controls.^20^ Furthermore, Gilbert et al.^23^ (2009) observed that the parental genotype presented a better agronomic performance than the transgenic lines resistant to SCYLV but higher infection rate with the virus. Therefore, these differences could be explained by the somaclonal variation generated during the *in vitro* regeneration process of the transgenic lines. Yao et al.^69^ (2017) observed that all the transgenic lines evaluated produced higher cane and sucrose yield per hectare, as well as lower incidence of SCMV disease compared to the parental variety at two cutting ages.

Regarding bacterial diseases, Zhang et al.^25^ (1999) transformed *albicidin* (*albD*) gene into sugarcane and generated the plants without leaf scald disease. To control fungal infections, the *β-1,3-glucanase* gene from *Trichoderma* spp. was transformed into sugarcane and the transgenic plants showed variable resistance levels against the fungus *Colletotrichum falcatum* that causes the disease known as red rot.^26^ In addition, Tariq et al.^27^ (2018) evaluated the transgenic sugarcane lines expressing a *class II barley chitinase* gene for protection against *C. falcatum*, and found the crude protein extracts from the transgenic plants inhibited mycelial growth under *in vitro* conditions.

Significant sugarcane productivity is damaged by attack of biological pests. One of the most important pest stresses that negatively impacts sugarcane production is caused by stem borers of the order Lepidoptera.^34^ Advances in plant genetic engineering have achieved broad protection against pests by incorporating genes from different organisms such as plants, pests, and bacteria^47,70–72^ (Table 1). Among the insecticidal proteins from plant organisms, we can mention lectins and protease inhibitors (PI). The genes from plant sources used to develop transgenic plants resistant to sugarcane crop pests are *avac* (*Amaranthus viridis* L. agglutinin gene), *skti* (Kunitz soybean trypsin inhibitor gene), *sbbi* (Bowman-Birk inhibitor gene) and *gna* (*Galanthus nivalis* agglutinin gene). Different studies have shown that they can suppress the growth, development, and reproduction of pests when they are expressed in high doses.^45,46,73^

Christy et al.^49^ (2009) evaluated the effect of sugarcane plants transformed with the *aprotinin* gene on *Scirpophaga excerptalis* larvae, observing a decrease in larval weight of up to 99.8%. Later, Schneider et al.^47^ (2017) also obtained good results using PI. They produced transgenic sugarcane lines that overexpressed the *CaneCPI-1* gene and found that they were resistant to *Sphenophorus levis* larvae compared to non-transgenic plants.

The main insecticidal proteins for obtaining transgenic plants are produced by the bacterium *Bacillus thuringiensis* (Bt). This bacterium produces proteins during its vegetative growth (*vip*) and sporulation phase (*cry*) that are highly toxic to a broad order of insects upon entering the alimentary tract.^74,75^ Therefore, their use has been spread to produce transgenic plants that are efficient against the target insects but not toxic to other non-target insects.^76^ Crops genetically modified with Bt genes have not only revolutionized the control of some of the main Lepidoptera and Coleoptera pests in important crops such as corn, soybeans, and cotton,^77^ but also been important for reducing the negative impact produced by harmful chemical insecticides in agriculture.^78^ The Bt transgenic plants resistant to stem borers have been successfully generated by use of these toxins for sugarcane cultivation. Numerous reports described different Bt genes (*cry1Ab, cry1Aa3, cry1Ac, s-cry1Ac, m-cry1Ac, cry2A*, and *vip3A*) under the control of CAMV 35S promoters and maize ubiquitin (maize Ubi-1) had been introduced into several genotypes of sugarcane (Table 1).

In 1997, transgenic sugarcane plants carried the *cry1Ab* gene under the control of the CAMV 35S promoter from the plant pathogen Cauliflower Mosaic Virus were obtained.^28^ The genetic transformation was accomplished by electroporation of intact cells. Transgenic sugarcane plants showed high resistance to larvicidal activity, even in the lines with low expression of *CryIA(b)*. Using the same gene, *cry1Ab*, Arvinth, et al.^29^ (2010) used Southern analysis to demonstrate the differences in the number of transgene inserts by the bioballistics and *Agrobacterium* transformation, and confirmed multiple transgene integrations in both the cases of particle bombardment and integration at a single site in *Agrobacterium*-mediated transformants in sugarcane. Islam et al.^30^ (2016) analyzed three *Agrobacterium*-transformed transgenic plants by Southern blot, and revealed that the transgenic plants had a few copies of the *Cry1A(b)* gene. In addition, they observed that the line with the lowest number of copies expressed 6–19 fold more of the transcript than the line with the most copies.

To generate genetically modified crops for commercial application, Wang et al.^11^ (2017) introduced the Bt insecticide gene *cry1Ab*, the glyphosate-tolerant gene *epsps*, and the *pmi* selection marker gene for phosphomannose isomerase into sugarcane through *Agrobacterium*-mediated transformation. The results revealed the excellent performance of the transgenic lines in terms of targeted traits. However, they exhibited poorer agronomic and industrial traits than non-transformed plants. Therefore, to achieve transgenic lines with potential for commercial use, it is necessary to transform a large number of explants. In a similar investigation, Gao et al.^41^ (2018) changed the sugarcane callus with *cry2A* gene and phosphinothricin acetyltransferase gene (*bar*) by particle bombardment. The *bar* gene used for selection also gives an agronomic characteristic of resistance to the Basta® herbicide. Unlike previous work, the transgenic plants not only showed resistance to the pest but also good agronomic and industrial traits comparable to non-transformed plants.

Another gene used in sugarcane engineering is *cry1Ac*. Several studies attempted to increase the percentage of GC bases in *cry1Ac* gene to favor the conformation of open chromatin and activate transcription in plants.^34,79,80^ The transgenic plants showed resistance to stem borer infestation under greenhouse and field conditions. Therefore, they demonstrated the potential of engineering new genes to increase the resistance level in the transgenic sugarcane plants.^34^ In a further study, *cry1Ab* and *cry2Ab* Bt genes with different mechanisms of action were stacked. The transgenic plants did not present stem borer damage and presented similar agronomic characteristics to their non-transformed parent variety. Furthermore, it showed that the high-dose expressing gene stacking strategy effectively prevents the generation of resistance in at least 100 generations of sugarcane borer, as long as it is combined with the refuge strategy.^43^

Riaz et al.^42^ (2020) developed transgenic sugarcane plants resistant to the *Chilo infuscatellus* pest by expressing the toxin gene *vip3A*. The insertion of a single copy of *vip3A* gene in the transgenic lines is sufficient for stem borer resistance. They also reported that these lines could generate the material for forming gene pyramids with other Bt toxins and thus prolonging resistance. The studies described above demonstrated that introducing Bt toxins by transgenesis in sugarcane effectively controls stem borers. Some work has resulted in commercial products. The first commercial GM sugarcane variety resistant to *D. saccharalis* was released in 2017, with two more varieties released in subsequent years. The transgenic events CTB141175/01A, CTC91087-6, and CTC93209-4 were commercially released in Brazil. Besides, the event CTB141175/01A was approved for commercialization in the USA and Canada, and the event CTC91087-6 was approved in the USA. All the three events express a toxin from the *cry* gene family, which confers resistance to Lepidoptera pests^81^ (Figure 2).

## Resistance to herbicides

Weed control in crop production is mainly chemical by use of broad-spectrum herbicides. However, prolonged applications are increasing herbicide resistance in many weeds. As this approach is expensive and eventually leads to increased herbicide loads in the environment, the development of genetically modified crops resistant to broad-spectrum herbicides is an alternative.^82^ On the other hand, traditional breeding for this trait is almost impossible due to the lack of herbicide-resistant genes in the genetics of wild relatives.^83^

We listed the most remarkable developments in incorporating herbicide resistance genes into sugarcane genomes in Table 1. Chowdhury and Vasil^7^ (1992) reported the first attempt to introduce herbicide resistance in sugarcane. They introduced a transformation vector with *bar* gene, a selectable gene that confers resistance to the herbicide Basta®, by biolistic and electroporation into suspension culture cells and protoplast. However, no whole sugarcane plants were obtained after the regeneration process of transformed cells due to the cell lines being old and non-morphogenic. Better results were obtained by Gallo-Meagher and Irvine,^8^ bombarding embryogenic calli with *bar* gene. In this case, field trials with glufosinate ammonium-resistant plants showed stable transgene expression in three rounds of vegetative propagation.

Moreover, the resistance was observed when the transgenic plants were propagated *in vitro* condition by meristem culture. Snyman et al.^9^ (1998) transformed embryogenic calli by the biolistic method using a plasmid harboring a synthetic *pat* gene, which also confers resistance to glufosinate-ammonium (Buster®). The regenerated transgenic lines showed stable expression of the herbicide-resistant gene during several ratoons. Also, the morphological and agronomic characteristics such as stalk height, diameter, population, fiber, disease resistance, and yield were not significantly different between the transgenic lines and the non-transformed plants in the first ratoon.^16^ Enriquez-Obregon et al.^15^ (1998) were able to obtain Basta®-resistant calli introducd *bar* gene by using *Agrobacterium*.

**Figure 1.**
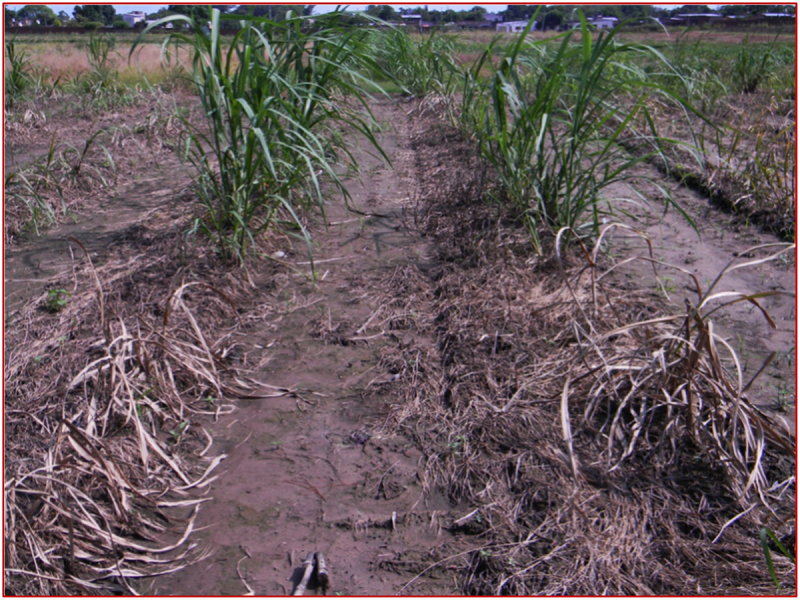
Field assessment of transgenic sugarcane events resistant to glyphosate herbicide. The figure shows the resistant transgenic sugarcane events compared with the non-transformed parental plants after glyphosate application. Photo courtesy: Dr. Aldo Noguera, EEAOC, Argentina.

The glyphosate herbicide (Roundup®) caused a revolution in the last century in weed control and crop production worldwide. Glyphosate is a broad-spectrum herbicide that inhibits the biosynthetic pathway of the aromatic amino acids, which are essential for protein synthesis and used as the precursors for hormones, lignins, and other defense compounds such as flavonoids and alkaloids.^84^ The success of glyphosate resistance has been demonstrated in various crops. However, only a few reports concerning glyphosate-resistant sugarcane have been found in the last decade. Nasir et al.^12^ (2013) transformed four sugarcane varieties by bombarding embryonic calli with a glyphosate-tolerant (GT) gene. After two rounds of glyphosate application, only the transgenic events with higher expression levels of the GT gene-encoded protein survived applications with high glyphosate concentration. It is worth mentioning that the transgenic event expressing the CP4 *epsps* gene from *A. tumefaciens* developed in Argentina by Noguera et al.^13^ (2015) was the closest attempt to release herbicide-resistant sugarcane worldwide (Figure 1). Exhaustive studies concerning health and environmental regulations were performed to assess their potential impact on agricultural systems and food safety necessary for commercial deregulation of any transgenic event in Argentina.^83^

**Figure 2.**
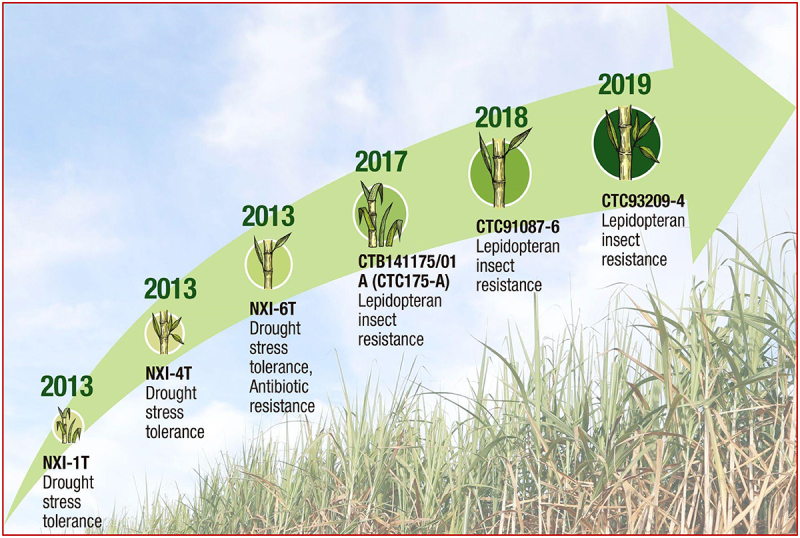
Transgenic sugarcane varieties released for commercial use around the world. The figure shows the sugarcane events approved for domestic or non-domestic cultivation.^81^

Gene stacking by genetic engineering allows the combination of different transgenes to increase resistance to insects and herbicides for improving crop productivity. Wang et al.^11^ (2017) reported the first transgenic sugarcane expressing the CP4 *epsps* gene for herbicide resistance and *cry1Ab* for pest resistance. As mentioned in a previous section, these transgenic events exhibited strong insect and glyphosate resistance under greenhouse and field growing conditions. Nevertheless, its agronomic and industrial performance was poorer compared to the parental control plants.

Nowadays, Genome Editing technology allows improving crops without delivering foreign genes into the plant genome. Recently, CRISPR/Cas9 was used for co-editing mutations of multiple alleles of acetolactate synthase (*als*) gene and recovering the herbicide resistance in sugarcane.^14^ More details concerning this work are shown in the Genome Editing section.

## Tolerance to abiotic stress

Sugarcane production is affected by abiotic factors globally.^3^ Many different genes have been used to mitigate the damage caused by these factors in sugarcane. However, there are still many challenges before the commercial release of transgenic varieties resilient to these stresses.^83^ The data in Table 1 show that it is mainly conducted under greenhouse conditions, so more studies on the field performance of a transgenic event are required to evaluate the possible effect on the stress tolerance capacity.

Deficient or excess water is the main factor affecting sugarcane productivity worldwide, and therefore there is a great interest in developing more water-deficient tolerant varieties in sugarcane producing countries.^85–87^ However, unraveling the complexity of plant responses to the water deficit and exploiting effective genes for developing more drought-tolerant varieties suitable for commercial crop production are still arduous tasks.^88,89^ The examples here described to develop water-tolerant plants are mainly based on the overexpression of transcription factors (TFs), responsible for the induction of water stress-induced genes, and the effector proteins for biomolecule stabilization. The first group of regulatory proteins associated with abiotic stress gene regulation in plants is the COR/DREB family.^90^ Reis et al.^53^ (2014) overexpressed *AtDREB2A* gene and enhanced drought tolerance in sugarcane. The transgenic plants exposed to water deficit under green-house growing conditions showed higher relative water content (RWC), carbon assimilation, sugar content, and bud sprouting without biomass damage. The B-box (*BBX*) proteins have significant functions regulating plant growth and development. Therefore, it also represents good candidate genes for enhancing stress responses in plants. Sugarcane plants overexpressing *AtBBX2*9 gene under water stress conditions showed higher photosynthesis rate and antioxidant and osmolyte levels.^56^ In another work, the overexpression of the tomato ethylene-responsive factor gene *TERF* in sugarcane caused an increased accumulation of osmolytes such as proline, soluble sugars, and glycine betaine. Moreover, the transgenic plants showed reduced malondialdehyde (MDA) content and reduced production of reactive oxygen species.^57^

The Arabidopsis H^+^-pyrophosphatase type I gene (*AVP*1) encodes a proton transmembrane transporter involved in the apoplastic pH regulation and auxin transport. Kumar et al.^51^ (2014) and Raza et al.^52^ (2016) reported overexpressing the *AVP1* gene transformed into sugarcane by biolistic and *Agrobacterium* methods. The transgenic plants showed enhanced drought and salinity tolerance as demonstrated by increased RWC, and osmotic and turgor potential in leaves of the transformed lines. Moreover, the transgenic plants showed increased root biomass (size, length) after stress. The pro-apoptotic BAX proteins regulate the programmed death cell (PCD) in plants.^91^ Ramiro et al.^54^ (2016) found that overexpressing a BAX inhibitor gene (*BI*-1) from *A. thaliana* in sugarcane plants enhanced the tolerance to drought by attenuating the induction of cell death pathways activated during water deficit.

Choline dehydrogenase enzyme participates in synthesizing glycine betaine, an osmoregulator that protects plants against cellular dehydration.^92^ It is noteworthy that Persero (PT Perkebunan Nusantara XI) developed the first commercially released drought-tolerant transgenic sugarcane expressing a bacterial choline dehydrogenase gene^81^ (Figure 2).

Under salinity conditions, plants accumulate compatible solutes such as proline, which act as an osmotolerant, also serve as a nutritional source, scavenge ROS, and preserve cellular functions. Guerzoni et al.^58^ (2014) developed a salinity-tolerant sugarcane event overexpressing pyrroline-5-carboxylase synthase gene (*P5C5*), whereas Li et al.^55^ (2018) enhanced the tolerance to water deficit to the crop, overexpressing a similar gene (*SoP5C5*). These P5C5 genes participate in proline synthesis.

Under abiotic stress conditions, plants need to eliminate the accumulated toxic molecules such as glyoxylate using ROS scavenging enzymes of the glyoxalase pathway [glyoxalase I (Gly I), glyoxalase II (Gly II), and glyoxalase III (Gly III)].^93^ The sugarcane plants overexpressing the EaGlyIII gene showed higher levels of RWC, photosynthetic pigments, osmolytes, and ROS scavenging enzyme activities compared to the non-transformed plants under high salt stress conditions.^60^ Heat shock proteins (HSPs) are a family of proteins produced by cells in response to exposure to stressful situations. Augustine et al.^59^ (2015) showed that the *E. arundinaceus* HSP70 overexpression in sugarcane plays a significant protective role in plants exposed to water deficit and salt excess. The transgenic plants showed higher stress-induced gene expression, cell membrane thermostability, RWC, and photosynthetic efficiency, etc.

Finally, limited reports about cold-tolerant transgenic sugarcane were mentioned in Table 1. The transgenic sugarcane overexpressing a bacterial isopentenyl-transferase gene (*ipt*) and using a cold-inducible promoter, At*COR15*a from *A. thaliana*, showed increased leaf chlorophyll content, reduced MDA accumulation, and electrolyte leakage concerning the non-transformed parental plants submitted to low temperatures.^61^ Chen et al.^62^ (2021) reported the transgenic plants overexpressing *α-tubulin* gene (*TUA*), which was cloned from a cold resistant sugarcane variety, improved the cold resistance of a cold susceptible sugarcane variety, showing higher contents of total soluble proteins and sugars, increased peroxidase activity, and lower MDA accumulation than the non-transformed plants under excessive temperature conditions.

## Genome editing

Genome editing is a type of genetic engineering in which DNA is precisely inserted, deleted, modified, or replaced in the genome of any organisms. Zinc-finger nucleases (ZFNs), transcription activator-like endonucleases (TALENs), and clustered regularly interspaced short palindromic repeat (CRISPR)/CRISPR-associated protein 9 (Cas9) system are the molecular tools that changed the horizon for plant genetics and biotechnology.^94^ Rapidly, GE was used to improve yields and nutritional quality of crops and also to increase abiotic tolerance and biotic stress resistance. Moreover, concerning other genetic engineering methods, edited crop plants are considered non-transgenic plants.^95^

However, due to the sugarcane genome complexity, only a few reports have been published for this crop.^83^ Altpeter and his group of scientists at the University of Florida led the first studies with nucleases to modify the sugarcane genome. Jung and Altpeter^96^ (2016) mutagenized the caffeic acid *O*-methyltransferase gene by TALEN nucleases. The mutant plants with altered cell wall composition showed a significant reduction in total lignin composition, which resulted in improved saccharification efficiency.^97^ Eid et al.^98^ (2021) used CRISPR/Cas9 to turn off 49 copies of the magnesium chelatase I subunit (MgCh) gene, a key enzyme for pigment biosynthesis, producing plants with severely reduced chlorophyll content. Afterward, Tufan Oz et al.^14^ (2021) showed the precise co-editing of multiple alleles of *als* gene induced by nuclease CRISPR/Cas9. The specific replacement of the target gene *via* template-mediated and homology-directed repair (HDR) of DNA double-strand breaks conferred herbicide resistance to the edited plants.

The Brazilian regulatory agency, National Biosafety Technical Commission (CTNBio), approved two non-transgenic varieties of genetically-edited sugarcane developed by Embrapa Agroenergy. The CRISPR/Cas9 edited sugarcane varieties, Cana Flex I and Cana Flex II, showed easier cell wall digestibility and higher sucrose concentration in plant tissues, respectively (https://www.embrapa.br/en/busca-de-noticias/-/noticia/66969890/ciencia-brasileira-desenvolve-primeira-cana-editada-nao-transgenica-do-mundo). Nowadays, the genome-edited plants are regulated as the transgenic in some countries but are considered not GM in others. The recent Brazilian approval is encouraging because the costs and efforts for a commercial release will be drastically reduced, and it is expected more countries will soon facilitate the deregulation of the varieties produced using this technology.

## Future prospects

Sugarcane varieties are the cornerstone of crop cultivation. The development of new sugarcane varieties remains one of the highest priorities for sugarcane agro-industries stakeholders around the globe. However, in the present dynamic era of climate change, access to a pipeline of newly developed varieties from one or more efficient biotechnological approaches will remain essential for the sustainability of sugarcane production around the globe. Furthermore, the CRISPR/Cas9 approach has emerged as an advanced technique for generating new varieties with specific desirable traits as various genes, beneficial or harmful for many important agronomical characteristics, can be manipulated easily. Although the impact of climate change on sugarcane is challenging to predict and is likely to be variable depending on the crop and environmental conditions, the overall view is that genomic engineering could contribute significantly to minimizing the impact of biotic and abiotic stressors on future sugarcane cropping systems. Genetic improvement of sugarcane through conventional breeding is increasingly complemented by molecular research to enhance the stress (biotic and abiotic) resistance, to maintain it as a major source of sugar and biorenewable energy.

## Abbreviations

*alb*D: *albicidin*
*als*: Acetolactate synthase
Bt: *Bacillus thuringiensis*
CP: Capsid protein
CRISPR: Clustered regularly interspaced short palindromic repeat
GE: Genetic editing
GT: Genetic transformation
GT: Glyphosate tolerant
HDR: Homology-directed repair
HSPs: Heat shock proteins
*ipt*: Isopentenyl-transferase
MDA: Malondialdehyde
P5C5: Pyrrolin-5-carboxylase synthase
PCD: Programmed cell death
PI: Protease inhibitors
*pmi*: Phosphomannose isomerase
RNAi: RNA interference
ROS: Reactive oxygen species
SCMV: Sugarcane mosaic virus
SCYLV: Sugarcane yellow leaf virus
ShRNA: Short hairpin RNAs
SrMV: Sorghum mosaic virus
TALENs: Transcription activator-like endonucleases
TERF: Tomato ethylene-responsive factor
TFs: Transcription factors
TUA: α-tubulin
ZFNs: Zinc finger nucleases
